# Editorial: Understanding the Processes Associated With Forgiveness

**DOI:** 10.3389/fpsyg.2020.628185

**Published:** 2020-12-21

**Authors:** Haijiang Li, Nathaniel G. Wade, Everett L. Worthington

**Affiliations:** ^1^Department of Psychology, Shanghai Normal University, Shanghai, China; ^2^Department of Psychology, Iowa State University, Ames, IA, United States; ^3^Department of Psychology, Virginia Commonwealth University, Richmond, VA, United States

**Keywords:** forgiveness, transgression, prosocial motivation, forgiving process, behavioral measurement

## Introduction

There has been a rapid growth in research on forgiveness over the past two decades. Although researchers have not reached a consensus on the definition of forgiveness (Worthington, 2020), there is general agreement that forgiveness is a changing process of prosocial motivation toward the transgressor, including changes in cognition, emotion, and motivation. Previous studies found that responding to offenses with forgiveness is associated with greater mental and physical health (Davis et al., 2015; Toussaint et al., 2015). Greater forgiveness is related to less neuroticism, depression, and rumination (Brown, 2003; Berry et al., 2005; McCullough et al., 2007), increased subjective well-being (Toussaint and Friedman, 2009), and improved interpersonal relationships (Riek and Mania, 2012). Individuals with lower levels of forgiveness also have higher levels of blood pressure, heart rate, and stress perception (Lawler-Row et al., 2011). Our understanding of forgiveness has increased remarkably with the breadth and depth of scientific research into many aspects of forgiveness. However, the process of forgiveness remains unclear. Due to the complexity of forgiveness, more research is needed to explore the process of forgiveness and the factors that affect the process. This current Frontiers Research Topic, brings together 10 articles that illustrate these questions, examining the process of forgiveness from different perspectives.

## Factors Affecting the Forgiveness Process

Hong et al. examined the influence of cognitive factors (e.g., compromising thinking) and personality traits (e.g., self-esteem) on forgiveness among a large adolescent sample. They found that compromising thinking forecasted decisional forgiveness rather than emotional forgiveness, and that self-esteem moderates the influence of compromising thinking on decisional and emotional forgiveness. Kong et al. found that rumination and anger play a mediating role between self-control and forgiveness. Using a behavioral measure of forgiveness, Liu and Li found that individuals with high self-control, expressed greater prosocial responses toward people who previously offended them, which is consistent with forgiveness. Ho et al. examined the facilitating role of emotional regulation on forgiveness and found that both self-regulatory strength and emotion regulation can predict forgiveness, and cognitive reappraisal plays a mediating role between self-regulatory fatigue and forgiveness. Moreover, previous studies found that empathy plays a vital role in facilitating forgiveness (Kimmes and Durtschi, 2016; Cornish et al., 2018). Ma and Jiang found similar results from a sample of adolescents.

## The Model of Motivated Interpersonal Forgiveness

To answer the questions of when, why, and how forgiveness occurs, Donovan and Priester (2017) proposed the Model of Motivated Interpersonal Forgiveness, in which they hypothesize that relationship closeness leads to a desire to maintain the relationship. This desire leads to motivated reasoning, which leads to interpersonal forgiveness. They found that motivated reasoning predicted forgiveness rather than empathy when controlling feelings for the other. In this new study, they replicated the abovementioned findings and excluded potential influences of the measures of motivated reasoning and the analytic estimation of hypothesis models (Donovan and Priester). The Model of Motivated Interpersonal Forgiveness suggests an explanation by which we can understand the psychological process of forgiveness.

## Forgiveness Over Time and Forgiveness Interventions

To explore the association between forgiveness and health, Long et al. examined the relationship between self- and divine- forgiveness and subsequent health and well-being using longitudinal data. They found that self-forgiveness and divine forgiveness were positively related to psychosocial well-being and negatively related to psychological distress. However, they found little evidence of associations of self- and divine- forgiveness with physical health or health behavior outcomes. Toussaint et al. examined the effectiveness of the REACH forgiveness psychological education method in a sample of Indian college students, which was effective with people of different religious commitments. Perceiving the offender as sharing a similar spirituality was associated with increased empathy, positive affect, and emotional forgiveness and less growth of unforgiveness as a result of the forgiveness training.

## The Repairing Effect of Apology and Compensation

Apology and compensation are methods whereby an offender attempts to repair relationship damage from transgressions by acknowledging their accountability. Offender behaviors were thought to promote the victim's empathy and forgiveness. Witvliet et al. found that apology and restitution each independently increased forgiveness and positive emotions while reducing unforgiveness, negative emotion, and muscle activity above the brow. Interactions observed greater effects of restitution compared to an apology, decreasing unforgiveness and anger while elevating positivity and gratitude. Komiya et al. conducted four studies to examine a socio-ecological hypothesis on apology and compensation. They found that compensation was more effective in appeasing residentially mobile people than stable people, while apology showed an opposite effect.

## Open Issues and Future Directions

This Frontiers Research Topic on “Understanding the Processes Associated with Forgiveness” displays many approaches to the study of the process of forgiveness, emphasizing the diversity of this topic, and paving the way for future research. However, there still exist numerous issues concerning forgiveness that need to be investigated. First, a better understanding is needed of the processes that govern an individual's decision to forgive and their experiences of emotional transformation. It is still unclear what the many influences on the process of forgiveness are. We also need to understand exactly how forgiveness impacts physical and mental health. Second, more effective methods are needed to describe the process of forgiveness besides self-report. Notably, when measuring forgiveness using behavioral measurements, different experimental tasks may reflect the different psychological processes associated with forgiveness. Whether the results based on different experimental tasks are comparable and reproducible, and whether simple behavioral responses can reflect advanced psychological processes needs further study. Finally, more research is needed on the cognitive neural mechanisms of forgiveness-related processes and the neural basis of forgiveness. We look forward to seeing this Frontiers Research Topic inspire exciting new research on forgiveness in a multi-faceted and integrated manner.

## Author Contributions

HL, NW, and EW conceptualized the article. HL drafted the manuscript. NW and EW substantively revised the manuscript, and read and approved the submitted version. All authors contributed to the article and approved the submitted version.

## Conflict of Interest

The authors declare that the research was conducted in the absence of any commercial or financial relationships that could be construed as a potential conflict of interest.
